# AlignNemo: A Local Network Alignment Method to Integrate Homology and Topology

**DOI:** 10.1371/journal.pone.0038107

**Published:** 2012-06-12

**Authors:** Giovanni Ciriello, Marco Mina, Pietro H. Guzzi, Mario Cannataro, Concettina Guerra

**Affiliations:** 1 Department of Information Engineering, University of Padova, Padova, Italy; 2 Department of Surgical and Medical Sciences, University Magna Graecia of Catanzaro, Germaneto, Italy; 3 College of Computing, Georgia Institute of Technology, Atlanta, Georgia, United States of America; Institute for Research in Biomedicine, Spain

## Abstract

Local network alignment is an important component of the analysis of protein-protein interaction networks that may lead to the identification of evolutionary related complexes. We present AlignNemo, a new algorithm that, given the networks of two organisms, uncovers subnetworks of proteins that relate in biological function and topology of interactions. The discovered conserved subnetworks have a general topology and need not to correspond to specific interaction patterns, so that they more closely fit the models of functional complexes proposed in the literature. The algorithm is able to handle sparse interaction data with an expansion process that at each step explores the local topology of the networks beyond the proteins directly interacting with the current solution. To assess the performance of AlignNemo, we ran a series of benchmarks using statistical measures as well as biological knowledge. Based on reference datasets of protein complexes, AlignNemo shows better performance than other methods in terms of both precision and recall. We show our solutions to be biologically sound using the concept of semantic similarity applied to Gene Ontology vocabularies. The binaries of AlignNemo and supplementary details about the algorithms and the experiments are available at: sourceforge.net/p/alignnemo.

## Introduction

In previous decades researchers have focused on the impact of the evolution at the genomic scale, i.e. how to reconstruct evolution by analysing genomic sequences. More recently, the availability of high-throughput data on protein-protein interactions allowed to look at evolutionary changes by comparing the map of interactions of proteins, also referred to as interactomes, of different species [1]–[3]. Goals of this field include the identification of conserved patterns of interactions among species as well as the identification of novel orthology relationships [4]. In this scenario several algorithms for the comparison of protein-protein interaction (PPI) networks have been developed, often referred to as network alignment algorithms.

The network alignment problem has two main instances: *global alignment* answers an evolutionary question by searching for a single comprehensive mapping of the whole set of proteins and protein interactions from different species; *local alignment* searches for evolutionary conserved building blocks of the cellular machinery, disregarding the overall similarity between networks. Formalism from graph theory provides the best framework to address both. Within this formalism, PPI networks are represented as graphs (*G*) whose nodes (*V*) are proteins and edges (*E*) are interactions among them. The protein network alignment problem is formulated as a graph alignment problem, i.e. the search for identical or similar subgraphs between two (pairwise) or more (multiwise) graphs. Formally: given two input graphs, 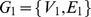 and 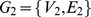, the problem of aligning 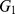 and 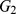 can be formulated as the problem of finding a mapping *M* between nodes in 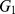 and nodes in 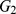 (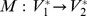, with 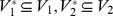) that maximizes an associated similarity function defined on nodes and edges. For the global alignment, M is the mapping between the whole sets of nodes of the networks. By contrast for the local alignment, M is defined as the set of mappings between the most similar subsets of nodes. In this paper we focus on local alignment of PPI networks and propose a method that aims at extracting conserved protein complexes in two PPI networks.

Protein complexes are here defined as groups of proteins performing similar function or involved in the same biological process. Existing approaches to detect protein complexes are generally based on the observation that complexes correspond to highly interacting sets of proteins and therefore they look for dense subgraphs in PPI networks. For instance, both versions of NetworkBLAST [5], [6] are based on such hypothesis, evolving from the initial PathBLAST [7] that focused on conserved paths. In our proposed approach, we look for *relatively* dense groups of nodes, i.e. nodes that have more interactions among themselves than with the rest of the network, imposing less rigid constraints on the topologies of complexes. Indeed, while the topology is informative, it has been shown to be often incomplete and reflecting a non-uniform knowledge over proteins [8], [9]. The presence of several false negatives leads to sparse graphs and even sparser sets of conserved interactions between species, causing approaches looking only for dense subgraphs to fail to detect conserved complexes.

Several approaches, such as NetworkBLAST, rely for the search of conserved complexes on a structure called *alignment graph*. The alignment graph has nodes corresponding to pairs of orthologous proteins and edges to conserved interactions. To cope with missing information, NetworkBLAST, as well as similar approaches, introduced less restrictive definitions of alignment graph, by allowing nodes to be connected if the respective pairs of orthologous proteins in the original PPI networks are at distance less than or equal to *k* (for NetworkBLAST 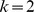). However, in this way several unreliable links may be added to the alignment graph leading to incorrect solutions even for small values of *k*.

The method Mawish [10] addresses network alignment as a maximum weight induced subgraph problem, incorporating evolutionary models to assess topological similarity. While effective, this model may be too strict leading, as we observed in our experiments, to identify only small conserved structures, and failing in recovering larger complexes.

Other algorithms, such as Graemlin [11] and its new version Graemlin 2.0 [12], generalize previous approaches by allowing the search of more general topologies. These methods increase the ability of detecting meaningful alignments by using, in addition to orthology information, paralogy relations between proteins from Inparanoid [13], KEGG pathway annotations [14], and known alignments. However, these approaches do not fully exploit topological information, as the local alignment step only examines the direct neighborhood of each node, grouping the best neighbors iteratively in a greedy fashion.

PHUNKEE [15] made a step forward in considering locally conserved subnetworks in a network context: after selecting sets of putative orthologs, this method explores all adjacent proteins at the same time looking for highly conserved interaction sets. However, all interactions have the same reliability and network context defined by PHUNKEE does not go beyond direct interactors. Finally, concurrently to the development of this work, a novel method, NetAligner [16], devised an algorithmic framework for the alignment of proteomes. NetAligner introduces a strategy to identify evolutionary conserved interactions, relying on the principle that interacting proteins evolve at rates significantly closer than expected by chance.

While a detailed description of available algorithms for both global and local network alignment is beyond the scope of this paper, a more extensive synopsis on available tools is provided in Table 1.

**Figure 1:**
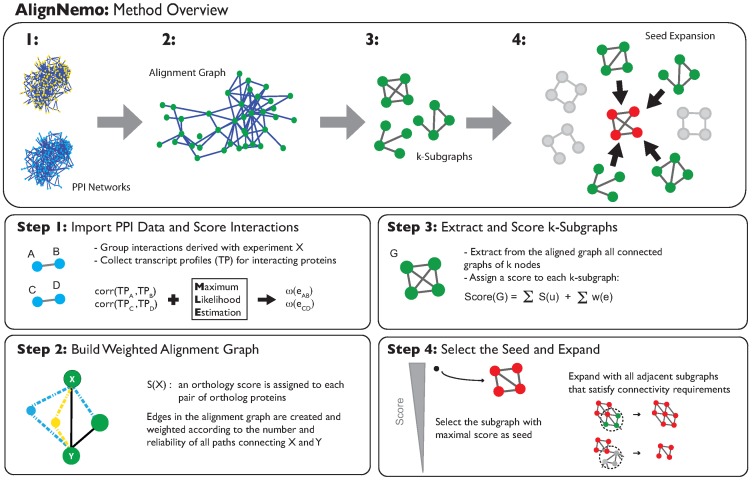
AlignNemo Overview. Given two input PPI networks (1) the alignment graph is built and scores are assigned to its nodes and edges (2). Then *seeds*, i.e. small subgraphs with a large number of high scoring links and nodes, are extracted from the alignment graph (3), and each seed is expanded in a greedy fashion, by adding small subgraphs that are relatively well connected to it by reliable links (4).

We introduce here a method, AlignNemo (Aligning Network Modules), that addresses the issues reported above by providing a general and effective framework for local network alignment. AlignNemo proceeds through different steps as outlined in Figure 1. First, it builds a weighted alignment graph from the input networks. Nodes represent pairs of putative orthologous proteins and are scored as in Inparanoid, reflecting the confidence on mapping the protein pairs. Edges, by contrast are weighted with a novel approach that accounts for the local connectivity in the input networks (see Methods). Then, we extract all connected subgraphs of a given size from the alignment graph and rank them according to weights on nodes and edges. Top ranking fully connected subgraphs will be used as seeds for the alignment solution. Finally, we expand each seed in an iterative fashion by adding multiple subgraphs at each step. This allows us to explore the network context of a solution beyond its immediate neighbors. A formal description of the algorithm is provided in the Methods section.

**Table 1:** A synopsis on network alignment tools.

Algorithm	Local(L) /	Pairwise(P) /	Input Data	Alignment Strategy*
	Global(G)	Multiwise(M)		
**Mawish** [10]	L	P	PPI Networks	Alignment Graph
			BLAST e-values	Single node expansion
				Duplication-divergence
				model
**PathBLAST** [7]	L	P	PPI Networks	Alignment Graph
			BLAST e-values	Single node expansion
				Conserved linear path
				extraction
**NetworkBLAST** [5]	L	P	PPI Networks	Alignment Graph
			BLAST e-values	Score for PPI reliability
				Single node expansion
				Conserved dense networks
				extraction
**NetworkBLAST-M** [6]	L	M	PPI Networks	Layered Alignment Graph
			BLAST e-values	Single node expansion
				Conserved dense networks
				extraction
**Graemlin** [11]	L	M	PPI Networks	Probability model to score
			Cluster of Orthologs	nodes and edges
				Nodes equivalence classes
				Single node expansion
**Graemlin** 2.0 [12]	G/L	M	PPI Networks	Machine learning approach
			KEGG Clusters	for network scoring
			Known Alignment	Single node expansion
**ISORANK** [33]	G	P	PPI Networks	Eigenvector of protein pair
				associations
			BLAST e-values	Consistent set of associations
				extraction
**ISORANK-N** [34]	G	M	PPI Networks	Greedy extension of ISORANK
			BLAST e-values	
**GRAAL** [35]	G	P	PPI Networks	Purely topology based
(see also [36], [37])			BLAST e-values	Protein pairs scored based on
				graphlet signature
**HopeMap** [38]	L	M	PPI Networks	Cluster of orthologs
			BLAST e-values	Alignment Graph
			Inparanoid Clusters	Strong connected component
			KEGG Clusters	extraction
**PHUNKEE** [15]	L	P	PPI Networks	Expansion process
			Metabolic networks	with addition of neighboring
			BLAST e-values, COG	modules
**NetAligner** [16]	L	P	PPI Networks	Interaction Conservation
			BLAST alignments	probabilities

* All methods, as a last step, score and rank the solutions according to a similarity function.

The main contributions of our approach are: 1) a new strategy to score the edges of the alignment graph that analyzes the structure of the input PPI networks through their collections of paths between two given nodes and estimates their reliability and local significance; 2) a new iterative expansion procedure, that starting from a seed, explores the local topology of the alignment graph at each step beyond direct interactions. This combination provides a new way to account for both topology and homology, and proves effective in detecting a large variety of protein complexes independently of their size or degree of connectivity.

In the next section, we show as a proof of principle results for the alignment of PPI networks of *S.cerevisiae*, *D.melanogaster*, and *H.sapiens*. We demonstrate that our alignments have superior topological and biological quality over other approaches. The quality of the results is evaluated in various ways: we first show the ability of AlignNemo to recover known protein complexes by means of the measures of precision and recall, then we show that our solutions are biologically sound using the concept of semantic similarity applied to Gene Ontology vocabularies, finally we show that the extracted modules preserve high connectivity even with the less restrictive constraints imposed by the method.

Representative complexes are discussed in details and comparisons are provided with local alignment tools such as NetworkBLAST, Mawish, and NetAligner as the only ones for which the software was available and currently maintained. We selected NetworkBLAST and Mawish for the main analyses as they are usable with user defined input data, while we compared AlignNemo and NetAligner separately as we ran the latter on its own data and interaction probabilities. AlignNemo, along with proper documentation and the datasets used in this paper, is available at http://www.bioinformatics.org/alignnemo.

## Results and Discussion

In this section, we evaluate the performance of AlignNemo, NetworkBLAST, and Mawish on publicly available datasets for *D. melanogaster* (fruit fly), *S. cerevisiae* (baker’s yeast), and *H. sapiens* (human). We ran these methods on the same datasets, and each algorithm produced a set of solutions, or modules, possibly overlapping. A module *M* is a subgraph of the alignment graph containing a set of protein pairs from the two input networks. We will refer to the set of proteins from network 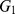 and 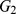 in *M* as 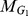 and 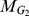 respectively.

Solutions from each method are evaluated and compared in terms homology and topology. First, we show that AlignNemo is able to recapitulate known protein complexes with high precision and recall. Then, we show that associations of proteins from different species are biologically sound using the concept of semantic similarity applied to Gene Ontology vocabularies. Finally, we prove that our solutions are more densely connected than expected by chance. We conclude this section by focusing on few specific cases to highlight weaknesses and strengths of each method.

### Input Data

Protein-protein interactions for *D. melanogaster* and *S. cerevisiae* were derived from the Database of Interacting Proteins (DIP - updated 10/27/2011) [17]. They include 7548 proteins and 22969 interactions in fly, and 5053 proteins and 22254 interactions in yeast. Inparanoid [13] was used to select 10045 pairs of putative orthologous proteins from the two networks, involving 1878 proteins from yeast and 1511 proteins from fruit fly. *H. sapiens* PPI network was derived from the HIPPIE database [18]; it includes 12113 proteins and 78559 weighted interactions coming from 17 different sources. A set of putative orthologous protein pairs from human and fly were obtained from the Gerstein Lab [19].

These data sets integrate multiple sources and include interactions derived from different methodologies including high-throughput and small scale experiments. To account for such diversity, we assign a reliability score to each edge. For both networks derived from DIP (fruit fly and yeast) we adopted the maximum likelihood estimation procedure defined in [20] to assess the reliability of protein interactions determined through the same experimental procedure. This method is based on the observation that correlations of gene expression profiles through different time points are good features to evaluate PPI reliability: interacting proteins typically show high correlation values. In applying this method we considered random pairs of proteins not known to be interacting as *true non-interacting* proteins, and interactions determined by small scale experiments as *true interacting* proteins, estimating from these two sets the respective distributions of correlation coefficients. For yeast proteins we used the set of expression profiles reported in the SGD database [21], and assigned a confidence score to each experimental method described in DIP and to combination of them. The scores of the fly interactions were computed based on the assumption that a given experimental method works equally well in different organisms and therefore the confidence scores based on yeast data were transfered to fly interactions. Reliability scores for the human protein interactions network were available through the web server HIPPIE.

### Detection of known Complexes

We assess the quality of the results by evaluating the agreement of the modules found by each method with known complexes. Given a module and a known complex, we compute two widely used measures from information retrieval: precision (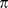) and recall (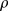). 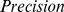 is defined as the percentage of proteins in the module that are also present in the complex; *recall* is defined as the percentage of proteins in the complex that are also present in the module. To integrate these measures into a single score, we compute the 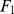-score function defined as the harmonic mean of precision and recall. Formally, these measures are defined as follow:
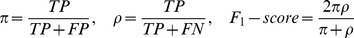
where *TP* is the number of true positives, i.e. the number of proteins found in a solution that are also in the complex. Analogously, *FP* and *FN* are the number of false positives and false negatives. The 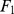-score ranges in the interval [0, 1], with 1 corresponding to perfect agreement. In our analysis, we match each known complex of species 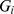 to all modules 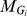 from a given algorithm and select as best match the module with highest 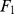-score.

**Figure 2:**
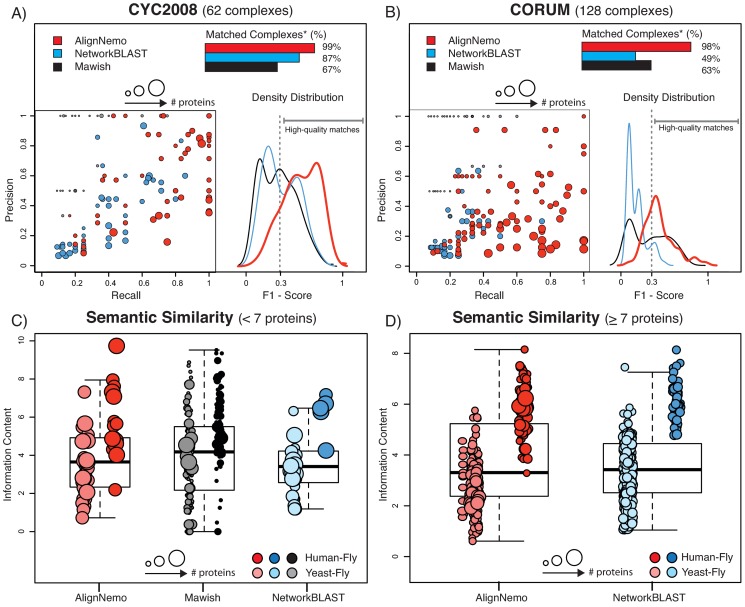
Comparison of AlignNemo, NetworkBLAST, and Mawish. The three algorithms are evaluated in terms of recovering known protein complexes in both 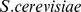 (CYC2008) and 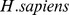 (CORUM). Solutions matching known complexes are scored by means of precison, recall, and F_1_ score. Obtained score distributions for each method are plotted in panel (A) for yeast-fly alignment, and panel (B) for human-fly alignment. Panels (C) and (D) show the average semantic similarity between proteins from different species mapped by each solution. Each solution is represented by a circle with the radius proportional to the size of the solution. The size of the solutions from each method varies significantly, thus small (<7 nodes) and big (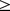 7 nodes) solutions are shown separately. * Percentages refer to the set of complexes matched by at least one method.

To evaluate the results for the alignment of *S. cerevisiae* and *D. melanogaster*, we referred to complexes in CYC2008 [22], which is a comprehensive catalogue of 408 yeast protein complexes derived from small scale experiments and literature mining. For the alignment of *D. melanogaster* and *H. sapiens*, we referred to complexes in CORUM [23], a dataset of 1682 human protein complexes. We observed that 28% of CYC2008 and CORUM complexes are composed by only 2 or 3 proteins (132 for CYC2008 and 474 for CORUM). This may be problematic as statistical measures tend to be hardly interpretable for such small complexes. For this reason, we restrict our analyses to complexes with at least 4 proteins, but at the same time we verified the ability of each method to recover small complexes (2–3 proteins). We considered a small complex to be recovered if at least 2 of its proteins overlap with an alignment solution, excluding the solutions exceeding 20 nodes. In Table 2 we summarize the performance of the four algorithms. In the table we list the number of modules found by each algorithm and, among those, the number of high quality modules, i.e. those that match a known complex with an 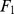-score greater than 0.3. The overall distribution of 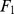-scores obtained by AlignNemo, Mawish, and NetworkBLAST is estimated by the respective kernel density distribution and shown in Figure 2 (A–B). In Figure 2 (A–B) we also report the performance of each method in terms of precision and recall separately. Both NetworkBLAST and AlignNemo perform better on the yeast-fly alignment, with the latter having overall higher values of both precision and recall. The small solutions found by Mawish have in general high precision while inevitably failing in recovering most proteins in a complex.

**Table 2:** Comparison of AlignNemo, Mawish, NetworkBLAST, and NetAligner.

	Fly-Yeast				Fly-human			
Algorithm	No. of S.	M.S.	F_1_>0.3	S.C.R.	No. of S.	M.S.	F1>0.3	S.C.R.
Mawish	175	32	29	16	87	37	60	33
NetworkBLAST	329	46	30	18	45	23	13	24
NetAligner	140	32	41	**49**	133	40	81	84
AlignNemo	242	54	**52**	27	115	53	**87**	**89**

**No. of S.**: Number of Solutions; **M.S.**: Matching Solutions; **S.C.R.**: Small Complex Recovered.

The number of solutions found by each algorithm (No. of S.) is listed in column 2 and 5 for the yeast-fly and the fly-human alignment, respectively. The number of solutions that match at least one known complex is reported in columns 3 and 6 (M.S. - Matching Solutions) for each alignment. The number of high-quality matches for complexes of size 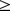 4 is summarized in columns 4 and 7 (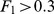), while the number of small complexes (2-3 proteins) recovered is in columns 5 and 8 (S.C.R. - Small Complex Recovered).

The complete lists of 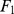-scores, along with the measures of precision and recall, are available as supplemental material (Table S1). For each match we also report the p-value derived by Fisher exact test after correction for multiple testing. AlignNemo clearly outperforms the other approaches in recovering known complexes, showing the highest percentage of high quality modules. It should be noticed that while Mawish performs similarly well for the fly-human alignment, the majority of modules produced by this method have small size, specifically 90% of them consists of 2 nodes only.

### Protein Mapping between Species

In the previous section, we showed that AlignNemo is able to recapitulate known protein complexes and that detected conserved sub-networks generally reflect known biology within each single species. On the other side, the quality of the mapping between proteins from different species needs further evaluation. We assess the biological relevance of the discovered mappings in terms of functional similarity, i.e. we determine to what extent the matched proteins from the two organisms are functionally related.

This analysis requires the use of prior biological knowledge that is encoded into ontologies. We choose the Gene Ontology (GO) framework and its annotations to determine the functional similarity between two proteins from different species, by using the concept of *semantic similarity* [24]. In our analysis, for each solution we computed semantic similarities using the set of annotations from the Biological Process (BP) and Molecular Function (MF) ontologies in GO. We report here results for BP only as this ontology more closely reflects the idea of protein complexes as sub-cellular units involved in specific processes. Complete results are reported in Table S2.

Given two proteins 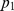 and 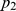, and their set of GO annotations 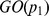 and 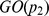, the Resnik similarity measure [25] is used to score each pair 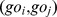 with 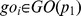 and 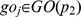. The semantic similarity of 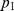 and 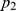 is defined as the average of the scores of the best match for each GO term in 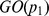 and 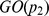 according to the Resnik measure [26]. Semantic similarities were computed using the tool FastSemSim [27].

In total we tested 356 solutions for AlignNemo, 85% of which have between 5 and 15 proteins and the largest 93 proteins; 362 solutions for NetworkBLAST, each including between 5 and 15 proteins, the latter being a limit imposed by the method; and 260 solutions for Mawish, each including between 2 and 6 proteins. Given the striking difference in terms of size of the detected sub-networks, we show the results obtained by the three methods separately for small complexes (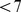 proteins) and large ones (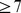 proteins) in Figure 2 (C–D).

Results for both protein network alignments show similar performance for the three algorithms in terms of semantic similarity, with better performance for the *H.sapiens* - *D.melanogaster* protein alignment.

### Topology of Conserved Modules

Here, we analyze the topology of the obtained solutions. As discussed in the Introduction, protein complexes are typically composed of densely interacting proteins. However, recent findings on modularity and organization of complexes in PPI networks show that they tend to consist of a densely connected *core* and a less strongly connected set proteins defined *attachment*. The latter is typically present in multiple complexes and allows diversification of potential functions [28].

Following this model, AlignNemo looks for *relatively* densely connected proteins, i.e. proteins that have more interactions among themselves than with the rest of the network, rather then imposing rigid and fixed constraints on the topology of a candidate solution.

We want now to test whether this strategy puts at risk our ability to detect densely connected cores, including among our solutions sparse sub-networks unlikely to be actual protein complexes. To this purpose, we generate 1000 random networks for each PPI network, preserving their node degree distribution; then we evaluate for each module its connectivity, i.e. number of edges, in the original PPI networks and in the random set. Thus, for each species and each solution, we estimate a background distribution of its connectivity. We quantify the deviation of the observed connectivity in the real network, 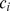, from such background distribution using a Z-score:
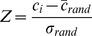
where 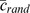 is the average connectivity for this set of proteins in the random set and 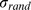 its standard deviation.

First, we test separately the two sets of proteins defined by each solution, one for each species, then, we associate to each solution the maximum Z-score between the two obtained. By this way, we account also for relatively poorly connected proteins in one species, when the corresponding orthologs in the other species are densely interacting. A p-value is empirically derived for each module from this background distribution, and it is given by the number of random networks that led to a greater or equal Z-score for the tested module over all possible networks. Interestingly, we found that 95% of the solutions, both for the human-fly and the the yeast-fly alignments, show statistically significant higher connectivity than those observed in the randomized networks.

In conclusion, AlignNemo outperforms both Mawish and NetworkBLAST in correctly detecting protein complexes within single species given their interactomes and orthology relationships. Furthermore, protein mappings between different species are biologically sound as proven by the average semantic similarity between proteins in the same module. Finally, despite AlignNemo does not impose rigid constraints on the module topology, exploring less strongly connected components of a protein complex, the extracted subnetworks are more densely connected than expected by chance.

### Comparison with NetAligner

NetAligner relies on a novel algorithmic approach to compute probalitities associated to conserved interactions, based on protein sequence similarity between proteins from different species. Given two pairs of putative orthologs, NetAligner evaluates the likelihood that they share a conserved interaction by considering the difference of evolutionary distances between the two orthologous pairs. We tested NetAligner under different configurations and input data, including the original proteomes and homologies provided with the tool. According to our analysis NetAligner achieves the best performance when using the *predict likely conserved interactions* setting, together with the parameters suggested in its reference paper [16]. NetAligner extracts a bigger and more reliable set of alignments on its own dataset. Therefore we decided to compare AlignNemo and NetAligner each run on its own dataset.

**Figure 3:**
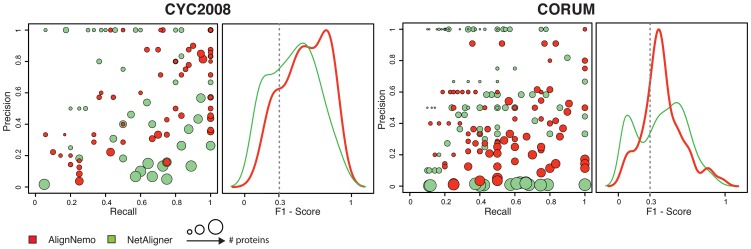
Comparison of AlignNemo and NetAligner. The two algorithms are evaluated in terms of recovering known protein complexes in both 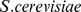 (CYC2008) and 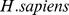 (CORUM). Solutions matching known complexes are scored by means of precison, recall, and F_1_ score.

When the solutions are matched to the reference complexes (CYC2008 and CORUM), the two methods perform similarly (see Figure 3 and Table 2). AlignNemo shows again better overall performance for the 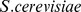-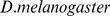 alignment. In the 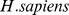-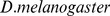 alignment, NetAligner finds a set of higher scoring small solutions, but at the same time several matches are produced by a very large solution including 463 nodes, leading to high recall values despite a precision close to zero (Figure 3).

### Conserved Complexes

In this section we focus specifically on few complexes of CYC2008 and CORUM to better dissect the performance of different methods. Cases discussed here include a small complex, *Arp2/3*, and two relatively large complexes, *TFIID (general transcription factor)* and *20S Proteasome*, with different level of connectivity. In Table 3 we report the proteins of these complexes that have been correctly asssociated and recovered by at least one between AlignNemo, NetworkBLAST, and Mawish in the 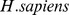 and 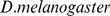 network alignment. For both the Transcription Factor TFIID and Arp2/3 complexes AlignNemo performs better according to both F_1_-score and semantic similarity. In detecting the 20S Proteasome, AlignNemo and NetworkBLAST have comparable recall for yeast-fly alignment, but AlignNemo has higher precision. Also, AlignNemo shows a superior performance in the human-fly alignment. Significantly enriched GO catagories for our solutions have been computed with GOTermFinder [29] and are reported in Table S3. In both alignments the cross-species semantic similarity is higher for AlignNemo indicating an improvement in biological quality, the details of which are discussed below.

**Table 3:** Comparison of the best matching solutions for Arp 2/3, TFIID, and 20S proteasome complexes.

Complex Name: Actin related protein 2/3 (ARP 2/3)					Complex size: 7 proteins			
Method:					Mawish	AlignNemo		N.BLAST
Solution size:					-	6		-
Protein Function	ID Human		ID Fly		Correctly selected			
ARP 3B	ARP3B		P32392			•		
ARP 2/3 subunit 2	ARPC2		Q9VIM5			•		
ARP 2/3 subunit 3	ARPC3		Q9VX82			•		
ARP 2/3 subunit 5	ARPC5		Q9VQD8			•		
**Complex Name: Transcription Factor IID (TFIID)**					**Complex size: 13 proteins**			
**Method:**					**Mawish**		**AlignNemo**	**N.BLAST**
**Solution size:**					**2**		**19**	**10**
Protein Function	ID Human		ID Fly		Correctly selected			
TFIID subunit 1	TAF1		P51123				•	•
TFIID subunit 1 like	TAF1L		P51123				•	•
TFIID subunit 10b	TAF10		Q9XZT7		•		•	
TFIID subunit 11	TAF11		P49906				•	
TFIID subunit 6	TAF6		P49847				•	•
TFIID subunit 7	TAF7		Q9VHY5				•	
TFIID subunit 8	TAF8		Q9VWY6		•		•	
TFIID subunit 9	TAF9B		Q27272				•	
TBP	TBP		P20227				•	•
**Complex Name: 20S Proteasome**					**Complex size: 14 proteins**			
**Method:**					**Mawish**		**AlignNemo**	**N.BLAST**
**Solution size:**					**2**		**11**	**11**
Protein Function		ID Human		ID Fly	Correctly selected			
Proteasome subunit alpha type-1		PSA1		P12881			•	•
Proteasome subunit alpha type-2		PSA2		P40301			•	•
Proteasome subunit alpha type-3		PSA3		Q9V5C6			•	
Proteasome subunit alpha type-4		PSA4		P18053			•	•
Proteasome subunit alpha type-5		PSA5		Q95083			•	
Proteasome subunit alpha type-7		PSA7		P22769	•		•	•
Proteasome subunit beta type-1		PSB1		P40304			•	
Proteasome subunit beta type-2		PSB2		Q9VQE5			•	
Proteasome subunit beta type-3		PSB3		Q9XYN7	•		•	
Proteasome subunit beta type-7		PSB7		Q9VUJ1			•	

Homologous proteins correctly included in the best matching solution of at least one algorithm. For Arp 2/3 complex, 4 out of 6 proteins really participate to Arp2/3 human complex, while the other 2 (omitted) are homologous proteins incorrectly included in the solution. NetworkBLAST and Mawish did not provide any solution overlapping with this complex. For TFIID and 20S proteasome complexes, the quality of AlignNemo solution is highlighted by the number of protein pairs belonging to the complex but not selected by Mawish and NetworkBLAST.

#### Transcription Factor TFIID Complex

RNA polymerases (I, II, and III) catalyze transcription of nuclear genes and rely on general transcription factors to recognize target promoters; in particular RNA polymerase II relies on the TFIID complex to initiate transcription. The general transcription factor TFIID is mainly composed of the TATA box binding protein (TBP) and a set of TBP-associated factors (TAF
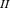
s) or subunits that are well conserved across species [30].

AlignNemo outperformed existing methods in uncovering this complex: it found 9 proteins of TFIID in a solution of 19 nodes; it correctly mapped human proteins into fly proteins corresponding to the same subunit in the two organisms (see Table 3). Mawish features a solution with only 2 nodes, also included in our alignment, while NetworkBLAST returned a solution of 10 nodes that match 4 proteins pairs belonging to TFIID complex.

Because of the high-connectivity of this complex, AlignNemo and NetworkBLAST solutions extend beyond the boundaries of TFIID complex as defined in CORUM. To further verify the quality of these solutions, we test all the proteins within them for enrichment of GO terms. We found that up to 16 out of 17 fly proteins and 18 out of 19 human proteins in AlignNemo’s solution are enriched for the same GO terms including *Transcription from RNA polymerase II promoter* (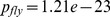, 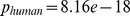). By contrast, the solution of NetworkBLAST reported only 4 out of 10 proteins in both networks with a common and specific biological role (see Table S3).

#### Arp2/3

Arp2/3 complex consists of 7 units and plays an important role in the regulation of the actin cytoskeleton. It is a major component of the actin cytoskeleton and is found in most actin cytoskeleton-containing eukaryotic cells [31].

Interestingly, the level of connectivity between these proteins in the original PPI network varies significantly, from 17 interactions found in human to none identified in 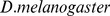. Incomplete information makes this complex particularly challenging to recover. Indeed, only AlignNemo was able to identify this conserved complex in 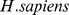 and 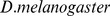, while both NetworkBLAST and Mawish did not have any solution in overlap with it. Table 3 lists the correctly detected homologous proteins that were found in the solution of AlignNemo. All 4 are annotated with the *regulation of actin filament polymerization function* GO term (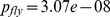 and 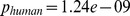). This case nicely points at the importance of considering conserved paths, rather than only direct interactions, to complement missing information in one network.

#### 20S Proteasome Complex

The 20S Proteasome is a large protein complex present in several organisms, in particular in all three organisms considered here. According to CYC2008 and CORUM, the 20S proteasome consists of 14 proteins in yeast and 16 proteins in both human and fly. The topology of the complex is relatively dense and the interactions are reliable.

For the case of 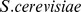-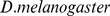 network alignment all three methods have comparable values of recall; as for the precision, NetworkBLAST obtains a much lower value since it finds several proteins outside the complex. On the other hand, AlignNemo outperforms the other methods in identifying the 20S Proteasome complex in the 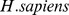-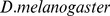 network alignment (see Table 3). Indeed, it correctly selected 11 proteins of the 20S Proteasome in human and 12 in fly, while NetworkBLAST found only 4 in human and 5 in fly and Mawish only 2 in both networks.

## Methods

AlignNemo aims at identifying protein modules or complexes that are conserved between PPI networks from different species. The search for conserved modules is performed on the alignment graph and consists of three major steps.

- First, the alignment graph is constructed from the input networks. Each node in the alignment graph corresponds to a pair of putative orthologous proteins, and scores from Inparanoid are used to weight each node. Each edge of the alignment graph is weighted according to a scoring strategy that incorporates information on the network context in terms of number, reliability and local significance of the paths connecting its endpoints in the input networks. This strategy is implemented by means of an auxiliary structure, the *union graph*, that is crucial to the overall performance of the method.
- Second, all connected *k*-subgraphs (here 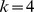) are extracted from the alignment graph and scored based on weights of nodes and edges. Top ranking fully connected *k*-subgraphs will be used as seeds for the alignment solution.
- Third, each seed is expanded in an iterative fashion by exploring the local neighborhood of the current solution beyond its immediate neighbors. Specifically, we define an expansion process that adds at each step all subgraphs that are more tightly connected by reliable interactions to the current solution than to the rest of the network.

This approach is in line with recent findings on modularity and organization of complexes in networks, according to which complexes in PPI networks tend to consist of a *core* part and *attachments*. The core is defined as a small group of proteins that are functionally similar and have highly correlated transcriptional profiles. The core is surrounded by less strongly connected proteins, defined attachments, present in multiple complexes which allow diversification of potential functions [28]. This diversification is well reflected by the structure of our solutions. Indeed, as shown in the previous sections, we identify several overlapping modules, rather the separated subnetworks with no intersection.

### Alignment Graph

The alignment graph 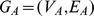 is a weighted graph, in which nodes represent pairs of homologous proteins and edges conserved interactions. As already mentioned, the existing definitions of the alignment graph differ in the way edges are set between two nodes. Most representations exploit a limited amount of topological information from the input since they discard almost all the nodes not involved in homologous associations and their interactions.

Our goal is to build an alignment graph that takes into account as much as possible the structure of the two networks. We designed a new scoring strategy for the edges of the alignment graph that incorporates topological information present in the original networks in terms of number, reliability and significance of paths of length less than or equal to 2 between two nodes. This strategy is best described and implemented by introducing an auxiliary structure that we call *union graph*. The construction and scoring of the alignment graph consists of three steps: (i) merge all input network data into the union graph, (ii) process the union graph to create a raw alignment graph, and finally (iii) perform some pruning operations on the raw alignment graph to remove noise and speed up the overall computation.

#### Union graph

The purpose of the union graph is to merge all input data into a single graph without losing information. Given two weighted networks 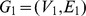 and 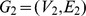, and a set of homologous associations 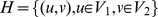 between the nodes of 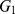 and 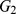, the union graph 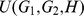 contains two type of nodes: (i) *composite nodes* representing pairs of homologous proteins, one from each network, as listed by *H*, and (ii) *simple nodes* representing the proteins of the two input networks that do not have an homolog in the other network. Any edge contained in one of the input networks is represented in the union graph by adding an edge between all pairs of corresponding nodes, either simple or composite. Formally:

**Definition 1.** The **union graph** 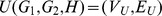 is a graph having the following structure:



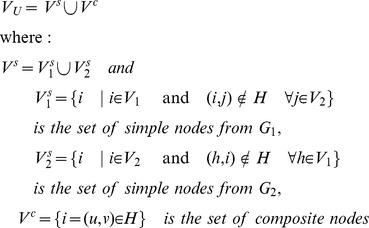





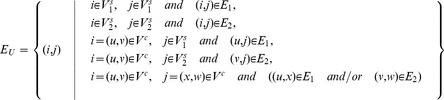



Assume that each edge *e* of 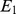 and 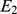 is labeled with a reliability score 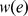, and each association 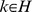 is labeled with a reliability score 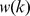. Then edge 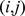 in 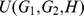 is assigned a score 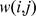 given by the score of the corresponding edge in the input network; the only exception is when both *i* and *j* are in 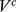, i.e. they are composite nodes, and there is a corresponding edge in both input networks, in such a case 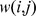 is the sum of the scores of the two original edges.

**Figure 4:**
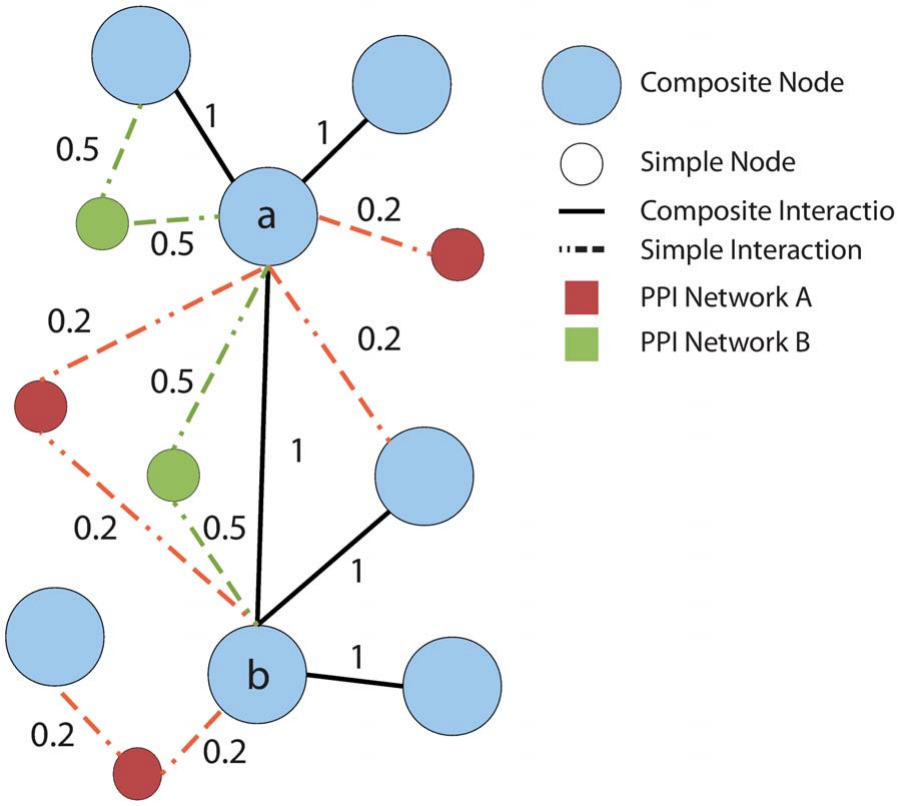
Example of union graph. The union graph includes both *composite* nodes representing pairs of homologous proteins from the two species (light blue nodes), and *simple* nodes representing the proteins that do not have an homolog in the other network (red and green nodes). Similarly, both composite interactions (black edges) and interactions present in only one species (red and green edges) are present in the union graph.

Figure 4 gives an example of the structure of a union graph.

#### Raw alignment graph

The alignment graph 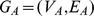 can be seen as a reduced version of the union graph in which only composite nodes are retained and an edge connects two nodes if there is at least one path of length less than or equal to 2 between the two nodes in the union graph. The intermediate node of a path of length 2 may be either simple or composite. The most important part of the definition of the alignment graph consists of an edge scoring strategy that summarizes the local topology of the union graph by taking into account all paths connecting two nodes in the union graph that satisfy certain criteria. This strategy is based on the assumption that homologous proteins connected by a large number of paths are likely to be functionally related. Each path between the two nodes thus provides additional evidence of their relatedness.

The choice of considering pairs of nodes at a distance not grater than 2 in the union graph appears reasonable. On the one hand, considering only directly connected node pairs is not suited for aligning evolutionary distant species and it is not robust against missing interactions in original PPI networks. On the other hand, adding edges between node pairs at a distance greater than 2 significantly increases the number of edges of the alignment graph, without providing any benefit in terms of quality of results, as our experiments showed. It has to be noted that some paths of length 2 in the union graph are spurious, i.e. they do not correspond to a path in an input network. Such paths are ignored in our analysis.

Paths of length 2, henceforth referred to as *indirect paths*, take a major role due to the missing interactions in the original PPI networks. However, not all the indirect paths have the same significance. In particular, indirect paths may pass through highly or loosely interacting proteins. If a node is highly interacting within the union graph then the probability that two nodes communicate through it is high. Moreover, the edges composing different paths could have different confidence scores and might represent conserved or non-conserved interactions.

To take all these observations into account we devised a new score based on Jaccard index [32]. Each edge 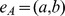 in the alignment graph is scored based on the number of paths of length 2 that link *a* and *b*. The final score of the edge between two nodes *a* and *b* of 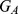 is given by the sum of two terms: a direct contribution 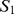 and an indirect contribution 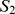. The direct contribution is evaluated as the ratio of the score of the direct path 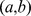 connecting *a* and *b* in the union graph (if it exists) divided by the sum of the scores of all the direct paths connecting *a* or *b* to any other composite node in the union graph. Analogously, the indirect contribution is evaluated as the ratio of the score of the paths of length 2 connecting *a* and *b* in the union graph divided by the sum of the scores of all the paths of length 2 connecting *a* or *b* to any other composite node in the union graph. Formally, we define this collection of paths connecting two composite nodes as their *extended local interactome* and derive a score as follow:

**Definition 2** - **Extended Local Interactome (ELI) score.** Let 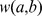 represent the score of the edge connecting nodes *a* and *b* in the union graph (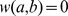 if 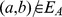) and 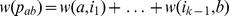 be the score of a path of length *k* connecting *a* and *b*. Then, if 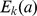 is the set of paths connecting *a* to its neighbors at distance *k*, and 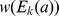 is the sum of the scores associated to these paths, we have:
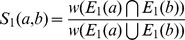


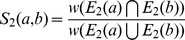


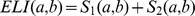



The power of this scoring strategy relies in its ability to account once again for the local neighborhood of aligned nodes: while methods such as NetworkBLAST or Mawish allow for gaps or mismatchs to connect conserved proteins at distance 2 in the aligned graph, we account for the whole set of paths connecting pairs of conserved proteins and for their reliability.

An example is presented in Figure 4 where for simplicity, we assume that each solid black edge has score 1, and each edge present only in the first or second network has a score of 0.5 and 0.2, respectively. Consider nodes labeled *a* and *b*. The direct path connecting *a* and *b* has score 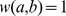. Node 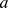 has 3 composite nodes connected through conserved edges, and 1 composite node connected through non-conserved edges. Node *b* has 3 composite nodes connected through conserved edges, and 0 composite nodes connected through unpaired edges. Therefore, the contribution of direct paths is:
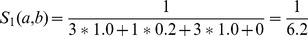



There are 3 indirect paths between *a* and *b* scoring respectively 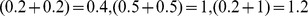. Node *a* has 6 indirect paths connecting it to other composite nodes, for a total score of 7.6. Node *b* has 7 indirect paths connecting it to other composite nodes, for a total score of 8.2. Therefore, the contribution of indirect paths between *i* and *j* is
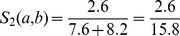



The final score is 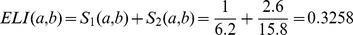.

#### Pruning the Union Graph

The alignment graphs resulting from the above construction tend to be very dense with edge scores spreading over a wide range of values. Removing less reliable edges is thus necessary for simplifying the alignment graph and reducing the computational cost in the next steps of the alignment procedure. Two interesting facts emerge when looking the distribution of edge scores:

- Few edges have a score significantly higher than the others.
- Edge scores vary considerably across different regions of the alignment graph and are affected by topological characteristics, such as interaction density. Thus, pruning the edges based on a global threshold may not be appropriate.

Following these two observations we designed a pruning strategy that processes all edges incident to the same node at once, and retains only locally high scoring edges. A simple yet effective rule has been used:

For each node 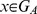, let 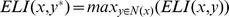. For a given constant *t*, all the edges 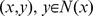, with score 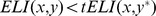 are deleted.

**Figure 5:**
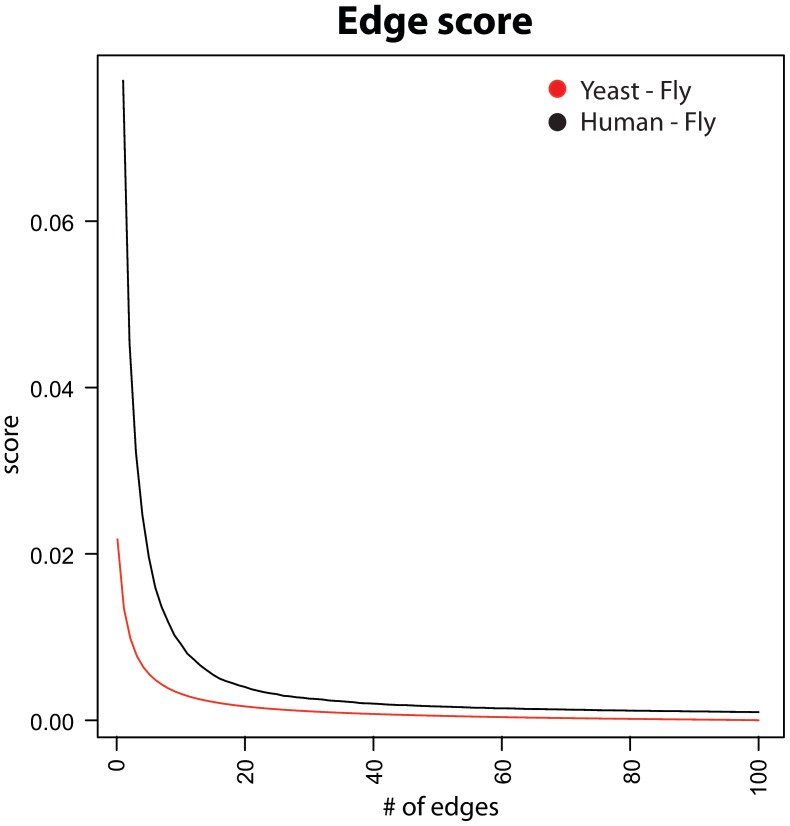
The edges incident to a node are ranked according to the their score. A value plotted on the curve is the average over all nodes of the alignment graph of the scores of the edges of the same rank incident to the nodes. To have comparable distribution of values, we select all the nodes on the union graph with at least 100 edges. The black curve corresponds to the human-fly alignment graph with 1578 nodes and the red curve to the yeast-fly alignment graph with 9325 nodes. Independent of the aligned networks, scores decrease exponentially making the pruning step both essential and effective.

This pruning strategy is tunable by varying the threshold *t*, thus allowing to create denser or sparser networks. In our tests we used 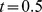. Pruning thresholds *t* ranging from 0.3 to 0.7 were tested with similar results. This was expected, since the distance between high scoring and low scoring edges incident to the same node is sharp, as clearly visible in Figure 5. On the other hand, not pruning low scoring edges (*t* = 0) introduce a huge number of spurious edges. Indeed, the application of this procedure leads to a drastic reduction of the number of edges of the alignment graph.

#### Dealing with multiple orthologs

Homology associations are tipically many-to-many and proteins associated to many putative orthologs will appear as multiple nodes in the alignment graphs. This becomes critical when such proteins are included multiple times within the same solution decreasing the accuracy on the final mapping.

We propose a strategy that exploits the topology of the networks to correct the weight of the edges connecting nodes involved in multiple homologous associations. Assume that 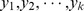, 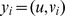, are nodes of 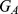 corresponding to multiple associations of the same node 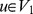, with *k* nodes 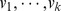 of 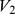. Furthermore, assume that 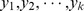 are all adjacent to node *x* in the alignment graph. We want to identify among these perhaps conflicting associations the ones that most likely correspond to true interactions with *x*. We sort the edges 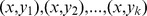 according to their score 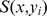 and denote by 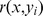 the rank of edge 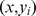 in the sorted list. Then we correct each score by dividing it by its rank:
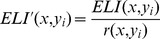



This correction reduces the weight of the edges leaving the highest scoring ones unaffected. We applied this procedure before the pruning of the edges as described above. We observed a significant improvement both in terms of the quality of the solution and computational costs. For simplicity, in the rest of the manuscript we will refer to this corrected score as 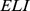.

Table 4 reports statistics on the alignment graphs produced for the 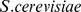-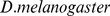 and 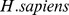-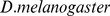 network alignments.

**Table 4:** Statistics on the size of the union graph and alignment graph.

	human - fly	fly - yeast
Union graph nodes	18535	19844
Union graph edges	51515	303341
Alignment graph nodes	1992	8809
(no multiple-ortholog correction)		
Alignment graph edges	3526	38789
(no multiple-ortholog correction)		
Alignment graph nodes	1941	5554
Alignment graph edges	2973	4740

For the alignment graph two cases are considered: when there is a correction of the weights assigned to edges due to multiple orthologs (as in our experiments) and when this correction is not applied.

### Seed Generation

A seed consists of a small subgraph of the alignment graph of fixed size *k*, i.e. a *k*-subgraph. First, all *k*-subgraphs are extracted from 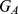, allowing arbitrary overlap of nodes and edges, then the non-overlapping top scoring ones are selected as seeds while the remaining will only be used to iteratively expand. We set 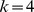 in all our experiments.

Enumerating all *k*-subgraphs with arbitrary overlap can be time consuming due to the large number of small subgraphs that is possible to extract even from sparse networks. To optimize the extraction process, we implement a simple heuristic to avoid counting the same instance multiple times, so that each subgraph is found exactly once. Precisely, we first impose an arbitrary order on the nodes of the graph 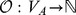, then we extract all subgraphs containing node *u*, by iteratively looking at nodes at distance less than *k* from *u* in the graph, 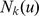, such that 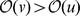, for each 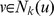.

We assign a score to each *k*-subgraph based on the individual scores of its components, i.e. nodes and edges. Precisely, given a subgraph *g* of the alignment graph 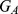 and denoted by 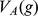 and 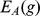 the set of nodes and edges of the subgraph *g*, respectively, we define:
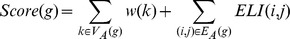
where 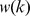 scores the confidence in the the two associated proteins being orthologous, and 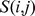 is the score of the edge 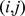 in the alignment graph as defined above.

### Module Discovery

Once all *k*-subgraphs have been extracted and scored, the algorithm ranks them according to their scores and selects the one with highest score as *seed*. Starting from the seed, the algorithm expands the candidate solution iteratively. The algorithm consists of a number of expansion steps. During each expansion step, all the *k*-subgraphs adjacent to the module, i.e. sharing at least one node with it, are considered as candidates for expansion. All the *k*-subgraphs that satisfy specific requirements are added to the module, thus at each step one or more *k*-subgraphs are added to the current module.

The selection of the *k*-subgraphs to add to the module is a key point of the method and we need to provide here few definitions. In the following, we denote by 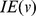 the set of edges of graph 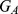 incident on node *v*, and by 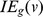 the set of edges of subgraph *g* incident on node *v*. Finally, for a subset *S* of *T* we denote by 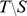 the subset of elements of *T* that are not in *S*. Given the current module *M*, a candidate subgraph *g*, and the remaining part of the alignment graph 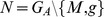, the set of edges incident on a node 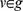 can be divided into subsets according to which subset the other endpoint belongs to, i.e. *g*, 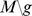, or *N*. Formally:
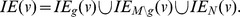



First, we define a *k*-subgraph *tightly* connected to the module if
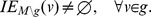



Tightly connected subgraphs are always added to the module. *Loosely* connected subgraphs are attached if they connect to the module with more reliable links than to the rest of the network.

Using the notation introduced above, for a given *k*-subgraph *g* we define:
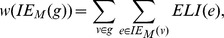


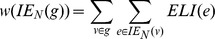
the sum of the weights of edges connecting *g* to the module, and the sum of the weights of edges connecting *g* to the rest of the network, respectively. Then *g* is added to the module if:



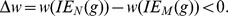



At the end of the expansion stage all accepted *k*-subgraphs are added to the module at once. The process is repeated until no more *k*-subgraphs can be added, thus we do not put upper limit on the size of obtainable complexes. On the other hand, we require our solutions to have at least 5 nodes, a limit imposed by the size of the seed (4 nodes) and the requirement of at least one expansion step to be completed. It is important to remark that expanding the module by *k*-subgraphs rather than by a single node at a time is not only crucial for the good performance of the method, but it is the key to account for multiple dependencies between a protein and its immediate neighbors.

### Implementation

AlignNemo is fully implemented in Java and has no dependencies from external libraries. The code and supporting documentation are available at: http://www.bioinformatics.org/alignnemo and a The alignment of S.cerevisiae and D.melanogaster required 3 minutes and 30 seconds, while the alignment of H.sapiens and D.melanogaster required 43 seconds. Both NetworkBLAST and Mawish are written in C, nonetheless the run-times we obtained are generally comparable to those of NetworkBLAST, while Mawish showed faster performance requiring 10 seconds for both the alignments.

## Supporting Information

Table S1 **Recovery of known complexes.** The table reports known yeast (CYC2008) and human (CORUM) complexes recovered by each method. Known complexes are identified by their ID, and for each complex the ID of the best matching solution for each method is reported. For each complex we report the total number of proteins, number of proteins in overlap with the best matching solution, precision, recall and F_1_-score.
(XLS)

Table S2 **Semantic Similarity.** The table shows the inter-species semantic similarity scores of the alignments found by each method. The semantic similarity is computed with respect to both the Biological Process and the Molecular Function vocabularies defined in Gene Ontology.
(XLS)

Table S3 **GO Enrichement Analysis.** Gene Ontology enriched categories of the solutions that best match the Arp2–3, TFIID, and 20S proteasome complexes.
(XLS)
